# Resistivity anisotropy of the misfit layer compound (SmS)_1.25_ TiS_2_

**DOI:** 10.1186/2193-1801-3-472

**Published:** 2014-08-27

**Authors:** Adel Nader

**Affiliations:** Department of Physics, Atomic Energy commission of Syria, P.O. Box 6091, Damascus, Syria

**Keywords:** Resistivity anisotropy, Misfit layer compounds, Lattice deformation, Magnetic ordering

## Abstract

**Background:**

The misfit layer compound (SmS)_1.25_ TiS_2_ has a layered structure which consists on an alternating sequence of one |SmS| bi-layer adopting a NaCl distorted structure and one |TiS_2_| tri-layer with the Ti atoms plane sandwiched by two sulfur ones and the Ti atoms octahedrally coordinated. Stacking is along the c-axis. Each of these two subsystems has its own 3D lattice and space group, and they have a common (b*, c*) plane in the reciprocal lattice so that the **a** axes are parallel and of irrational ratio. The physical properties of this compound have never been investigated. Accurate resistivity anisotropy measurement down to the liquid nitrogen temperature is presented here.

**Findings:**

The in-plane resistivity shows an anomaly at 202 K, and the out-of-plane resistivity shows also anomalies at 202 and 222 K and an upward curvature between 100 and 222 K. Consequently, the resistivity anisotropy shows peculiarities; mainly an important jump at 222 K.This behavior is discussed in the framework of a possible magnetic ordering and/or anisotropic lattices distortions. Although, the role of this work is to draw the attention to these features and further investigations to confirm these results would be of interest.

**Conclusion:**

Further investigations are needed, mainly magnetic and crystallographic in function of temperature in order to conclude definitely whether there is any phase transition or not.

## Introduction

The general formula of the misfit layer compounds family is (MX)_1+x_(TX_2_)_m_ , with M: transition metal or a rare earth, T: Nb, Ta, Ti, V ,Cr, X:S, Se, m = 1,2 or 3 and x = 0.08-0.28. Their structure consists on an alternating sequence of one |MX| quasi-cubic bi-layer with m |TX_2_| tri-layer sandwiches with a similar structure as that of dichalcogenides, stacked along the **c** axis. Each of these two subsystems has its own 3D lattice and space group. The relation between the two sub-lattices is given most briefly by a common (b*, c*) plane in the reciprocal space (Rouxel et al. 1995) so that the **a** axes are parallel and of irrational ratio.

The structure of the misfit layer compound (SmS)_1.25_TiS_2_ was resolved in (Cario et al. 1997) and except a rapid in-plane resistivity measurement the physical properties of this compound have not yet been investigated. Though, the combination of anisotropic structure with the mismatch along the **a-**direction as well as the rare earth element may induce interesting properties.

In this work, the in-plane and out-of-plane resistivities of (SmS)_1.25_TiS_2_ are determined down to the liquid nitrogen temperature. They both show peculiarities, and as a result the resistivity anisotropy shows an unusual behavior, mainly an important jump at 222 K.

The in-plane resistivity of (PbS)_1.18_(TiS_2_)_2_ (Meerschaut et al. 1992), which has a similar structure, is also determined for comparison. It shows an anomalous behavior above 200 K. Though, the sample size of this compound does not allow an out-of-plane resistivity measurement.

Our results are also compared with those obtained on other misfit layer compounds as well as on other transition metal dichalcogenides.

No definite explanation can be advanced, mainly because our results should be confirmed on other samples, but some indicators, based on possible magnetic ordering and/or anisotropic lattice distortions are discussed.

The role of this work is then just to draw the attention to the observed features and further investigation would be of interest.

### Experimental

Single crystals of (SmS)_1.25_ TiS_2_ are prepared as explained in (Cario et al. 1997). They appear as thin platelet that the thickness is in the range of 50 μm and the diameter of 1 mm. The **c-**axis is along the thickness. At least one sample of the same batch was analyzed by X-ray to determine the structure, although, such analysis cannot be performed on samples intended for transport measurements which often have a diameter in the range of 1 to 2 mm. Also, it has been well established for all dichalcogenides as well as for other misfit layer compounds that the **c-**axis is along the thickness.

**Figure 1:**
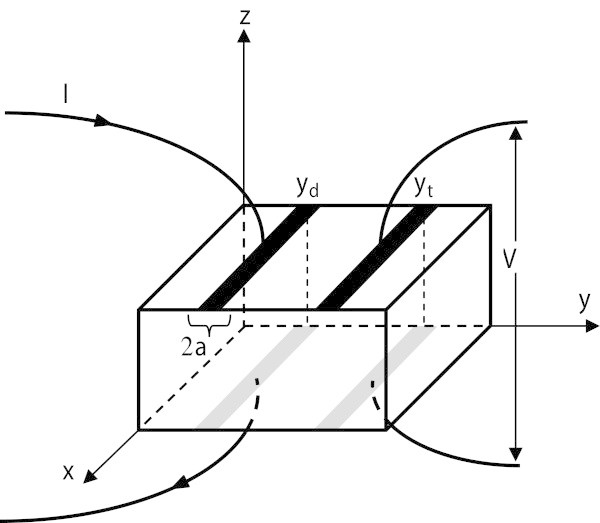
**Contacts Configuration.** Contacts configuration for the transverse resistance measurement. Note that contacts are extended along the sample width.

The in-plane resistivity is first measured by a regular four probe Montgomery method. Contacts made with silver paint are then washed and two contacts, extended along the sample width are put at each face, as shown in Figure 1. The transverse resistance is then measured down to the liquid nitrogen temperature, as in (Nader 2006).

Let *ρ*_*ab*_ and *ρ*_*c*_ be the in-plane and out-of-plane resistivities. *b*, *L* and *D* are the sample dimensions along *ox*, *oy* and *oz* respectively. The crystallographic **c-**axis is along the z-direction, and *Γ* = (*D*/*L*)(*ρ*_*c*_/*ρ*_*ab*_)^1/2^ is the so-called effective anisotropy.

*Γ* is extracted for each temperature by trial and error using our new formula of the transverse resistance, convenient for small *Γ* (Nader and Kouba 2010) , which can be written under the form:
1

with *α* = *πa*/*L*, *δ* = *πy*_*d*_/*L* and *γ* = *πy*_*t*_/*L* . 2*a* is the contact width along *oy* (see Figure 1) *δ* ∨ *γ* = max(*δ*, *γ*) and *δ* ∧ *γ* = min(*δ*, *γ*).

It is shown in (Nader and Kouba 2010) that *R*_*c*_ can be expressed by a quickly converging series, that the convergence is accelerated when the effective anisotropy approaches zero, and it is proved that the first term of the series is sufficient to express the resistance for *Γ* < 1.

The trial and error consists on varying *Γ* around an expected value in order to minimize the difference between the measured and calculated values of the ratio *R*_*c*_/*ρ*_*ab*_.

In order not to miss peculiarities during the measurement, liquid nitrogen cryostat is used for refrigeration, since it allows obtaining very slow temperature variations as the latent heat of liquid nitrogen is much greater than that of liquid helium.

Since the center of discussion of this work is the resistivity anisotropy, error bars are included on its plot. The main source of uncertainty is *R*_*c*_, although, special precautions are taken to avoid noise, mainly no pumps were used for refrigeration in order to avoid any phonic noise and at each measurement the nano-voltmeter was reset to zero and the measurement was averaged on 10 values, the uncertainty is then at its minimum.

## Results and discussion

**Figure 2:**
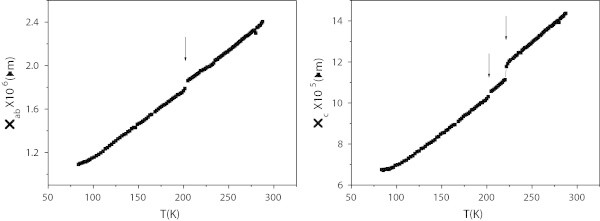
**Resistivities of (SmS)**_**1.25**_ **TiS**_**2**_ **.** **(a)** In-plane and **(b)** out-of-plane resistivities of (SmS)_1.25_TiS_2_. Arrows indicate anomalies.

Figure 2a and b show *ρ*_*ab*_ and *ρ*_*c*_ respectively, they are both metallic. *ρ*_*ab*_ is in the range of 10^− 6^*Ωm*, the same as other misfit layer compounds and shows a drop at about 202 K. *ρ*_*c*_ shows, as well, two drops at 202 and 222 K.

**Figure 3:**
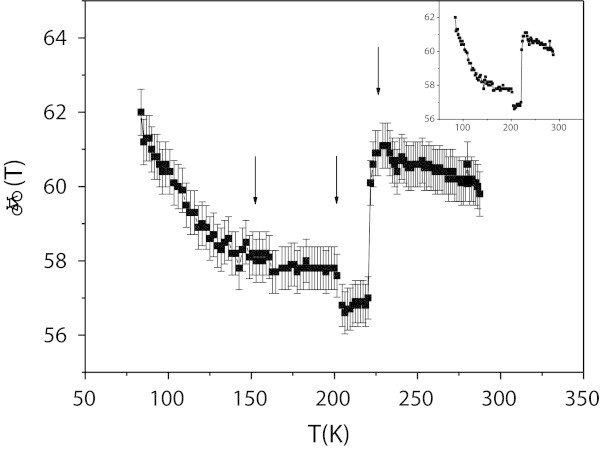
**Resistivity anisotropy of (SmS)**_**1.25**_ **TiS**_**2**_ **.** Resistivity anisotropy of (SmS)_1.25_TiS_2_. Note the jump at 222K. Raw data, without error bars is shown in the inset.

Let *β*(*T*) = *ρ*_*c*_/*ρ*_*ab*_ be the resistivity anisotropy. As shown in Figure 3, it shows two discontinuities at 202 and 222 K, and a smooth slope change at about 150 K, similar to that observed in 2*H*-NbSe_2_ (Leblanc and Nader 2010) at approximately the same temperature. An unusual aspect in *β*(*T*) is that it decreases when the temperature is raised and at 222 K it jumps to a value the same as at 90 K. The amplitude of this jump is about 92% of the whole variations of *β*(*T*) on our temperature range.

On the other hand, the values of *β*(*T*) (between 56 and 62) are relatively small compared to those of 2*H*-NbSe_2_ and 2*H*-TaSe_2_ (Leblanc and Nader 2010), as well as to the Bi-based misfit layer compounds (BiS)_1.11_(NbS_2_) and (BiSe)_1.10_(NbSe_2_) (Nader 2006) on the same temperature range. Though, it is of the same range as 1 *T*-TiSe_2_ (Nader and LeBlanc 2013) and 1 *T*-VSe_2_ (Nader et al. to be published). This low resistivity anisotropy seems then to be related to the (Ti,V) elements and probably to their octahedral coordination into the |TX_2_| layer.

**Figure 4:**
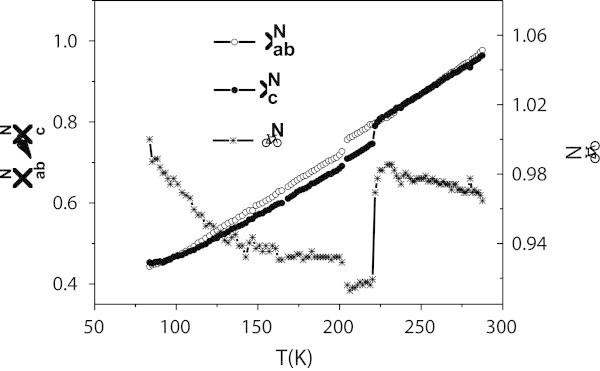
**Normalized Data Comparison.** ,  and *β* ^*N*^, which are *ρ*_*ab*_, *ρ*_*c*_ and *β* normalized to their maximum values of measurement, sketched on the same curve. Note that  shows an upward curvature between 100 and 222K.

*ρ*_*ab*_, *ρ*_*c*_ and *β* are sketched in Figure 4, normalized to their maximum values of measurement. This sketch reveals that *ρ*_*c*_ shows an upward curvature between 100 and 220 K when its slope rejoins again that of *ρ*_*ab*_ (Note that above 220 K both normalized curves of *ρ*_*ab*_ and *ρ*_*c*_ coincide, which means that they have the same slope since they are normalized to their maximum).

Let us compare our results with those obtained on other misfit layer compounds. In (Wiegers et al. 1991) the out-of-plane resistivity of (SmS)_1.19_(TaS_2_) shows a jump at about 70 K and an anomaly at 200 K, though, the method this resistivity is determined was not precised. The in-plane resistivity of this compound does not show any peculiarity. Also, the in-plane resistivity of (SmS)_1.25_(VS_2_) shows an uncommon behavior (Kondo et al. 1992); it varies metallically between 15 K and room temperature, with two slope changes at about 40 and 220 K, and varies in a semiconducting way below 15 K.

**Figure 5:**
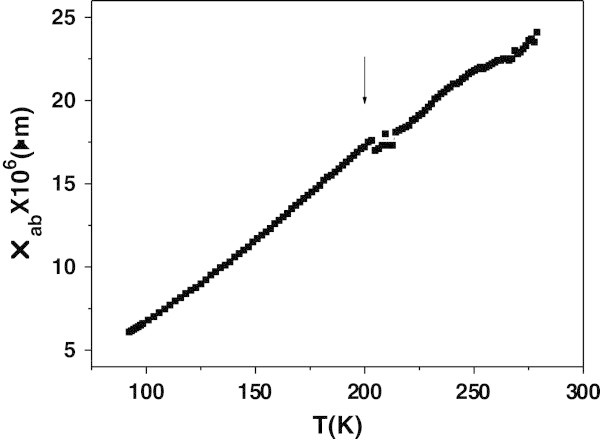
**Resistivity of (PbS)**_**1.18**_ **(TiS**_**2**_ **)**_**2**_ **.** The in-plane resistivity of (PbS)_1.18_(TiS_2_)_2_. Note the anomalous behavior for *T* ≥ 200.

Figure 5 shows the in-plane resistivity of the misfit layer compound (PbS)_1.18_ (TiS_2_)_2_ down to the liquid nitrogen temperature, it shows an anomalous behavior for T ≥ 200 K. Note that *ρ*_*ab*_(*T*) is a smooth curve up to 200 K and for higher temperatures it begins to show some irregularities, which means that these irregularities have a physical origin and not the result of noise. Though, since this compound has a similar structure than (SmS)_1.25_TiS_2_ it is questionable whether this structure is related to any change at temperatures above 200 K.

The same resistivity anisotropy measurements were done on the Bi-based misfit layer compounds (BiS)_1.11_NbS_2_ and (BiSe)_1.10_NbSe_2_ on the same temperature interval (Nader 2006) and no anomalies were noticed; the resistivity anisotropy decreases smoothly when the temperature is raised.

Also, the same behavior was observed in 2H-NbSe_2_ and 2H-TaSe_2_ (Leblanc and Nader 2010) as well as in some high T_c_ superconductors such as Bi_2_Sr_2_CaCu_2_O_8+δ_ doped with Fe, Co and Ni (Chen et al. 1999) or Bi_1.95_Sr_1.65_La_0.4_CuO_6+δ_ (Jin et al. 1995).The monotonic decrease in function of temperature seems then to be the most common for layer compounds. Although, there is no definite theoretical confirmation of this behavior, just some explanations concluding that it may result from differences in the scattering processes parallel and perpendicular to the conducting planes.

The origin of the in-plane resistivities' anomalous behavior seems then to be related to the |TX_2_| (T:Ti, V) sandwich for the misfit layer compounds.

On the other hand, the resistivity anisotropy of 1 *T*-VSe_2_ shows a minima similar to that found in (SmS)_1.25_TiS_2_ (Nader to be published).

Probably, the resistivity anisotropy behavior has as origin a magnetic ordering accompanied by an interaction with transport electrons, in a similar mechanism as Kondo effect, since similar misfit layer compounds (containing rare earth elements) show a variety of magnetic behaviors (Pena et al. 1991, Lafond et al. 1992).

Another possibility is anisotropic lattice distortions which influence the scattering modes parallel and perpendicular to the conducting planes. Anomalies in the in-plane resistivity curve of (PbS)_1.18_ (TiS_2_)_2_ would support this statement since this compound has no magnetic element.

As an indicator, in 1 *T*-VSe_2_ the lattice distortions were studied in function of temperature both along the **c**-axis and in the (**a**,**b**) plane (Tsutsumi 1982) , and an abrupt change in the **c-**axis was observed at about 85 K. Also, in (van Landauyt et al. 1978) a succession of incommensurate superlattices was observed in the same compound much above 110 K in an electron diffraction study.

Although, the role of this work is just to draw the attention to the interest that may manifest the properties of this compound, rather than to conclude definitely about its properties by a simple transport measurement on only one sample.

A definite explanation of the resistivity anisotropy behavior of (SmS)_1.25_TiS_2_ would first need to confirm the obtained results on other samples, and also magnetic and crystallographic investigation in function of temperature would be of interest.

## Conclusion

The resistivity anisotropy as well as the in-plane and out-of-plane resistivities of the misfit layer compound (SmTi)_1.25_ TiS_2_ are determined down to the liquid nitrogen temperature. The resistivity anisotropy shows notably a jump at 222 K. Our results need to be confirmed on other samples, and also further investigations mainly magnetic and crystallographic in function of temperature would be of interest.
